# Quality Characteristics and Clinical Relevance of In-House 3D-Printed Customized Polyetheretherketone (PEEK) Implants for Craniofacial Reconstruction

**DOI:** 10.3390/jcm9092818

**Published:** 2020-08-31

**Authors:** Neha Sharma, Soheila Aghlmandi, Shuaishuai Cao, Christoph Kunz, Philipp Honigmann, Florian M. Thieringer

**Affiliations:** 1 Department of Oral and Cranio-Maxillofacial Surgery, University Hospital Basel, CH-4031 Basel, Switzerland; neha.sharma@usb.ch (N.S.); shuaishuai.cao@unibas.ch (S.C.); christoph.kunz@usb.ch (C.K.); 2 Medical Additive Manufacturing Research Group (Swiss MAM), Department of Biomedical Engineering, University of Basel, CH-4123 Allschwil, Switzerland; philipp.honigmann@ksbl.ch; 3 Basel Institute for Clinical Epidemiology and Biostatistics, Department of Clinical Research, University Hospital Basel, CH-4031 Basel, Switzerland; soheila.aghlmandi@usb.ch; 4 Hand Surgery, Cantonal Hospital Baselland, Rheinstrasse 26, 4410 Liestal, Switzerland; 5 Department of Biomedical Engineering and Physics, Amsterdam UMC, University of Amsterdam, Amsterdam Movement Sciences, Meibergdreef 9, 1105 AZ Amsterdam, The Netherlands

**Keywords:** additive manufacturing, 3D printing, craniofacial, customized, dimensional accuracy, fused filament fabrication, patient-specific implants, PEEK, point-of-care, reconstruction

## Abstract

Additive manufacturing (AM) of patient-specific implants (PSIs) is gradually moving towards in-house or point-of-care (POC) manufacturing. Polyetheretherketone (PEEK) has been used in cranioplasty cases as a reliable alternative to other alloplastic materials. As only a few fused filament fabrication (FFF) printers are suitable for in-house manufacturing, the quality characteristics of the implants fabricated by FFF technology are still under investigated. This paper aimed to investigate PEEK PSIs fabricated in-house for craniofacial reconstruction, discussing the key challenges during the FFF printing process. Two exemplary cases of class III (Group 1) and class IV (Group 2) craniofacial defects were selected for the fabrication of PEEK PSIs. Taguchi’s L9 orthogonal array was selected for the following nonthermal printing process parameters, i.e., layer thickness, infill rate, number of shells, and infill pattern, and an assessment of the dimensional accuracy of the fabricated implants was made. The root mean square (RMS) values revealed higher deviations in Group 1 PSIs (0.790 mm) compared to Group 2 PSIs (0.241 mm). Horizontal lines, or the characteristic FFF stair-stepping effect, were more perceptible across the surface of Group 1 PSIs. Although Group 2 PSIs revealed no discoloration, Group 1 PSIs displayed different zones of crystallinity. These results suggest that the dimensional accuracy of PSIs were within the clinically acceptable range; however, attention must be paid towards a requirement of optimum thermal management during the printing process to fabricate implants of uniform crystallinity.

## 1. Introduction

Craniofacial reconstructions are often time consuming and present a significant challenge for the surgeon. A typical application is a cranioplasty, which is a standard neurosurgical procedure performed to reconstruct cranial defects. The critical clinical challenges in the reconstruction of craniofacial bone defects are the ability to carry out complex reconstructions with precise implant fit and esthetic appearance. The cumulative understanding of the surgeon to overcome these clinical challenges have led to the utilization of prefabricated patient-specific implants (PSIs) for cranioplasty procedures [1,2,3]. 

Over the past several years, polyetheretherketone (PEEK), a high-performance biopolymer, has gained substantial popularity in craniofacial reconstructions [4,5]. PEEK is an aromatic polymer with ether and ketone bond linkages. It is a high-temperature, semicrystalline thermoplastic material, which is chemically inert with high thermal stability and mechanical properties. Combining the characteristics intrinsic properties of PEEK, such as no artifact in medical imaging along with cortical bone-like modulus, it is an excellent alternative for metallic biomaterials in craniomaxillofacial reconstructive surgeries [6,7,8].

To date, the fabrication of PEEK implants is well matched to computer-aided design and computer-aided manufacturing (CAD/CAM) machining technologies like injection molding and, specifically, milling [9]. However, the recent advent of additive manufacturing (AM), popularly known as three-dimensional (3D) printing, is providing a replacement frontier for the design and production of prefabricated PEEK implants [9]. Schmidt et al. [10] first proposed AM of PEEK parts using the selective laser sintering (SLS) printing technology. Later, based on SLS technology, the EOS P800 (EOS, Electro-Optical Systems GmbH, Krailling, Germany) system was launched, which concentrated on AM of parts from PEEK powders at high temperatures [11]. However, the EOS P800 requires expensive PEEK powders, and the concentrated laser beam restricts the sintering process in extensive areas [12].

Compared to SLS technology, material extrusion-based fused filament fabrication (FFF) technology is already integrated into the hospitals for the fabrication of anatomical biomodels, customized surgical tools, and prosthetic aids [9]. Besides, recent technological advancements in FFF 3D printers have made it possible to process high-temperature PEEK thermoplastic biomaterial [13]. In the FFF process, a filament is continuously extruded from a heated nozzle in a viscous state and deposited in a layer-by-layer manner to form the desired shape of an object. FFF allows the fabrication of specific complex geometries that is not feasible by other manufacturing techniques such as milling or injection molding [9,14]. Unlike SLS, FFF offers numerous advantages, including low initial machine purchase costs, ease of use, less waste generation, and reduced risk of material contamination [15,16]. Considering these aspects, some FFF 3D printers are being explicitly developed for medical PEEK applications [17]. This technology currently contributes to a new point-of-care (POC) workflow, implementing how PEEK medical implants need to be designed, developed, and manufactured for low-volume and on-demand production. Previous studies have demonstrated the feasibility of using FFF for medical PEEK printing [13,17]. As this technology is recognized as a prospective tool in the medical sector for in-hospital manufacturing of customized PEEK implants, the extent to which it affects the geometry and manufacturing quality remain under investigated. Therefore, to implement POC manufacturing, an understanding of the relationship between the process parameters and the quality characteristics of the printed parts is crucial.

This paper aimed to analyze PEEK customized implants fabricated at the POC for craniofacial reconstructions, discussing the numerous challenges during the extrusion AM process. Furthermore, dimensional characteristics of the extrusion-based anatomically shaped PEEK cranial plates are reported for the first time in this paper.

## 2. Experimental Section

**Figure 1:**
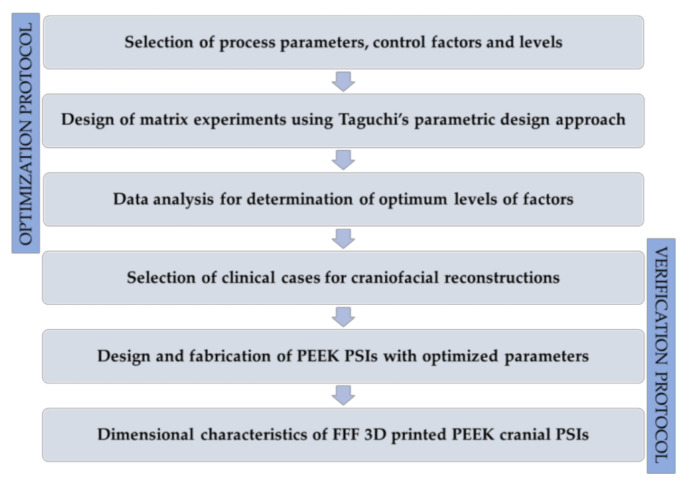
An overview of the schematic representation of the optimization and the verification protocols.

We present an investigation on the influence of printing process parameters on the dimensional accuracy of FFF 3D printed PEEK PSIs in this section. The entire experimental section was established using a procedural methodology, including two protocols, each involving several steps. Figure 1 displays an overview of the schematic representation of the optimization and the verification protocols.

### 2.1. PEEK Filament and FFF PEEK 3D Printer

For the printing process, the filament used was a medical-grade 1.75 mm PEEK filament (Evonik VESTAKEEP ^®^ i4 G resin, Evonik Industries AG, Essen, Germany). This unreinforced filament is a natural-colored, high-viscosity material that is specially designed for long-term implantable medical devices. This grade is often used for material extrusion-based technology. It has a density of 1.30 g/cm^3^ and a melting temperature of 380 °C. The PEEK filament was dried at 80 °C for 12 h in a filament drying unit (Apium Filament Dryer, Apium Additive Technologies GmbH, Karlsruhe, Germany).

The FFF 3D printer used was a desktop FFF PEEK 3D printer (M220, Apium Additive Technologies GmbH, Karlsruhe, Germany). The printer is equipped with a hot-air filter system designed to maintain a sterile printing environment following biocompatibility standard guidelines (ISO 10993) for an in-house or cleanroom POC manufacturing. The M220 is a third-generation Apium series printer with a closed-loop temperature management system with specific sensors, thermistors, and thermocouples to control the processing temperature. The technical specifications of the M220 third-generation series FFF PEEK 3D printer are displayed in Table 1.

### 2.2. Optimization Protocol for FFF PEEK 3D Printing Process Parameters

#### 2.2.1. Design of Experiments

Taguchi method is considered a valuable tool that provides a systematic methodology to optimize various design criteria [18]. The advantages of the design of experiments (DOE) using Taguchi’s technique are the simplification of the experimental plan and the feasibility to study interactions between various process parameters [19]. This method proposes an experimental plan using a particular set of arrays called orthogonal arrays. These standard arrays stipulate the way of conducting the minimal number of experiments that provide distinct combinations of parameters and their levels for each experiment. The protocol is especially vital for PEEK 3D printing, where the cost to produce prototypes is considerably high due to the expensive PEEK medical-grade material. Therefore, this parametric design approach was used to optimize the process variables for improving the dimensional characteristics. 

The investigated nonthermal processing parameters selected in this study were the layer thickness, infill rate, number of shells, and infill patterns. Layer thickness is the thickness of the material deposited by the nozzle at the successive layer during the printing process. Infill rate is the amount of material that is deposited inside the object. The number of shells is the number of perimeters printed on each layer of the object, and the infill pattern is how the filament is deposited inside the object. The selected four parameters, each at three levels, are illustrated in Table 2.

Based on the number of factors, levels, and the calculated degrees of freedom, the L9 Taguchi orthogonal array method was employed. Table 3 illustrates the experimental plan using an L9 (3^4^) orthogonal array.

#### 2.2.2. Fabrication of PEEK Test Objects

**Figure 2:**
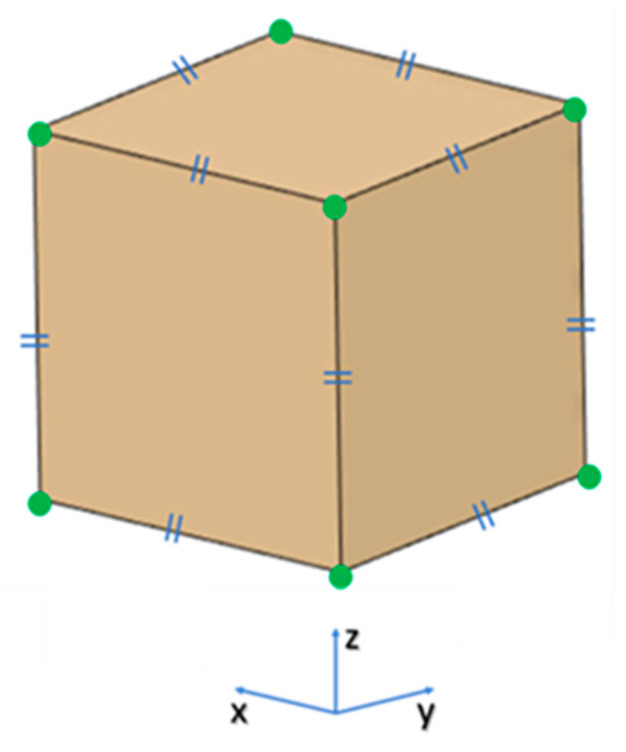
Projected view of a test object showing the landmark points: green points represent the landmark points for measurements and blue signs represent that the test object has equal dimensions (20 mm) in *x*-, *y*-, and *z*-axes.

The optimization protocol investigated the influence of four nonthermal printing process parameters (Table 1) on the dimensional accuracy of 3D printed PEEK samples. A test object measuring 20 mm × 20 mm × 20 mm, was designed using CAD modeling software (3-matic medical 13.0, Materialise, Leuven, Belgium) and exported in a Standard Tessellation Language (STL) file format (Figure 2).

Using the L9 orthogonal array (Table 3), nine samples were fabricated. To minimize experimental error, each sample was manufactured individually in the center of the printer’s build platform. After fabrication, each sample was measured with a high-precision electronic micrometer (Digital Micrometers Ltd., Sheffield, United Kingdom); accuracy: ±0.001 mm. Twelve linear measurements were taken for each sample concerning the printing axis, i.e., *x*-, *y*-, and *z*-axes. To enhance the experimental reliability of the statistical analysis and eliminate the interference of experimental error, each measurement was conducted three times. The percentage change in the dimensions [20] of the printed test sample was calculated using the following Equation (1):

(1) $$\%\;\mathrm{CD}=\left(\frac{x_{m}-x_{r}}{x_{r}}\right)\times 100,$$

where $x_{m}$ is the mean measured value, $x_{r}$ represents the reference CAD value, and CD stands for the percentage change in the dimensions of the test object.

### 2.3. Verification Protocol for Dimensional Characteristics of FFF 3D Printed PEEK PSIs

To evaluate the dimensional accuracy and clinical relevance of FFF 3D printed PEEK PSIs in craniofacial reconstructions, clinical cases were selected. The verification protocol encompassed several steps. It is described in the following sections.

#### 2.3.1. Data Acquisition and Computer-Aided Design (CAD) Modeling of Cranial PSIs

**Figure 3:**
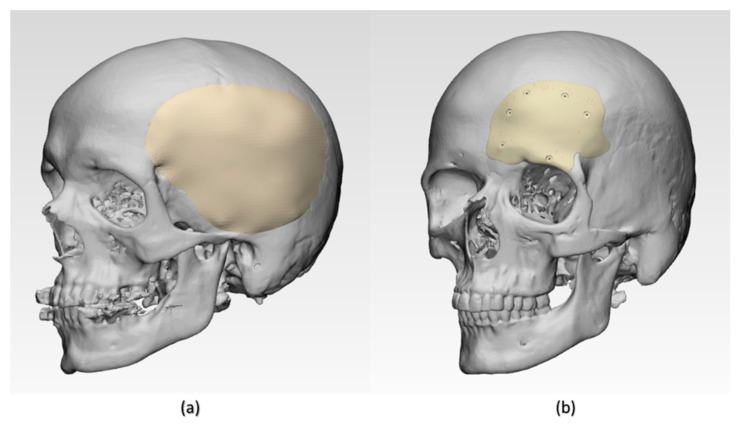
Illustration of three-dimensional (3D) volumetric reconstructions of craniofacial structures with patient-specific implants (PSIs). (**a**) Group 1 class III cranial defect reconstruction with PSI and (**b**) Group 2 class IV cranial defect reconstruction with PSI.

Two exemplary anonymized cases of craniofacial defects were selected from the hospital’s database. Ethical approval and patient consent were not applicable. The case selection was based on the classification of cranial implants on the degree of complexity in computer designing and manufacturing [21]. The selected craniofacial cases were categorized as class III and class IV cranial defects. Group 1 was a class III cranial defect case, representing a unilateral defect with a size larger than 100 cm^2^. Group 2 was a class IV unilateral cranial defect with orbital involvement, of a size larger than 5 cm^2^ and smaller than 100 cm^2^. High-resolution computed tomography (CT) scan images (Siemens SOMATOM, Siemens Healthcare GmbH, Erlangen, Germany) with a slice thickness of 1 mm were acquired in the exemplary cases. The Digital Imaging and Communications in Medicine (DICOM) datasets were then imported into Materialise Interactive Medical Image Control System (MIMICS) medical image processing software (MIMICS Innovation Suite v. 21.0, Materialise, Leuven, Belgium). Using a thresholding-based segmentation protocol for hard tissues, 3D volumetric reconstructions of the patient’s skull were generated. For the precise reconstruction of the craniofacial defect, a medically certified CAD modeling software was used (3-matic medical 13.0, Materialise, Leuven, Belgium). The mirroring function was used to replicate the corresponding contralateral healthy anatomical skull region. A surface reconstruction algorithm was then applied to generate the overall shape of the respective PSI (Figure 3). The CAD file of each PSI was converted into a 3D surface mesh and saved in an STL file format.

#### 2.3.2. FFF PEEK 3D Printing and Digitization of Cranial PSIs

**Figure 4:**
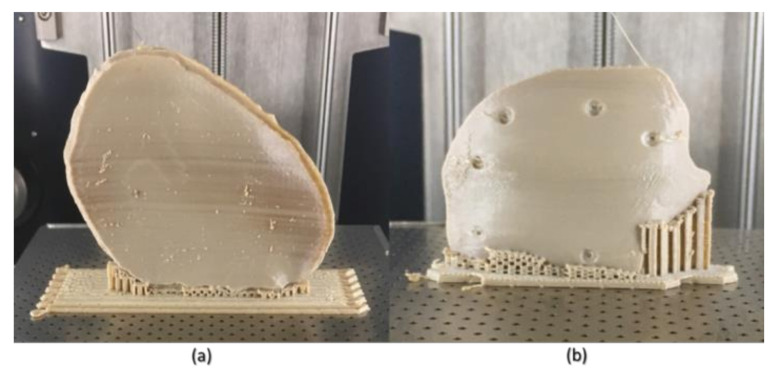
Fused filament fabrication (FFF) 3D printed polyetheretherketone (PEEK) cranial PSIs (in situ). (**a**) Group 1 PSI and (**b**) Group 2 PSI.

The STL files of the PSIs were imported into the 3D printer’s slicing software (Simplify3D 4.0, Cincinnati, OH, USA), and the fabrication of PEEK PSIs (*n* = 3 per group) was completed using the optimized process parameters (i.e., layer thickness, infill rate, number of shells, and infill pattern). To minimize experimental error, each PSI was fabricated in the center position of the build platform (Figure 4). After printing, postprocessing procedures were done to remove the support material. To generate an accurate point cloud representation of the 3D surface meshes, the fabricated PEEK PSIs were digitized using an optical-based scanning system (EinScan-SE, SHINING 3D Tech. Co., Ltd., Hangzhou, China). The 3D point cloud data generated by scanning the fabricated PEEK PSIs were then converted into an STL file format using an automated triangulation algorithm.

#### 2.3.3. Registration Protocol of the 3D Surface Meshes

For each cranial defect case, registration of the 3D surface mesh of digitized 3D printed PEEK PSIs to the 3D surface mesh of virtually designed PSI was done (3-matic medical 13.0, Materialise, Leuven, Belgium). First, superimposition (*n*-point registration) was performed with manually controlled points. Next, the surface-based global point registration function was used to superimpose datasets in the following steps: planned PSI (as a fixed 3D entity) and printed PSI (as a floating 3D entity) were subjected to best-fit alignment tool; calculation parameter options selected were: distance threshold, number of iterations, and subsample percentage. The distance threshold for registration was set higher than the average distance error. The number of iterations was defined until an average distance error of 0.1 mm was achieved. This registration protocol used the iterative closest point (ICP) algorithm to adjust the position of the floating entity automatically by superimposing it over the fixed entity. 

#### 2.3.4. Dimensional Accuracy Assessment of FFF 3D Printed PEEK Cranial PSIs

To evaluate the deviations and assess the accuracy of fabricated PEEK cranial PSIs, a 3D part comparison analysis was done (3-matic medical 13.0, Materialise, Leuven, Belgium). This comparison analysis function integrated into the software uses an ICP algorithm to calculate the closed point distance between the surface triangles of 3D surface meshes (planned and printed). A color-coded surface distance map was generated, which quantified the measurements as mean, median differences (positive and negative deviations), standard deviation, and root mean square (RMS). These color difference images were used to examine the qualitative congruence or incongruence between planned and printed PEEK cranial PSIs. The algorithm of the software matched and automatically calculated the deviations between the closest point pairs. The value of RMS was calculated by using the following Equation (2):

(2) $$\mathrm{RMS}=\sqrt{\frac{1}{2}\overset{n}{\underset{i=1}{\sum }}x_{i}^{2}},$$

If point A in planned PSI surface mesh has the closest point A’ in (digitized) printed surface mesh, then X_n_ is the distance between A and A’ and *n* is the total number of point pairs in both 3D surface meshes. The RMS value represented the overall 3D deviations and served as a measurement indicator of how far the deviations vary from zero between the two datasets.

### 2.4. Statistical Analysis

To analyze the experimental results of the optimization protocol, signal-to-noise (S/N) ratios, and analysis of variance (ANOVA) statistical methods were used. S/N ratio (η) determines the effect of each of the process parameters on the desired values and measures the variation of response to the target value. The objective of the optimization protocol was to minimize the percentage change in the dimensions. Thus, the S/N ratio (η) was calculated using smaller-the-better quality characteristics. The mean square deviation (MSD) was used to incorporate the effect of changes in mean and variation (standard deviation) with equal priority. The S/N ratio (η) was calculated using the following Equation (3):

(3) $$\eta =-10\;\log\;\left(\mathrm{MSD}\right)$$

where η is the S/N ratio and MSD is the mean square deviation for the output characteristics. To predict the optimum parameter level settings, the main effect plot for S/N ratio was used. To determine the effect of individual parameters along with their interactions, ANOVA was used. The level of significance was set at *p* < 0.05. This section of statistical analysis was performed in Minitab statistical software (Minitab 19, State College, PA, USA).

To analyze the results of the verification protocol for dimensional accuracy assessment, descriptive statistics were used. To summarize, the qualitative characteristics of PEEK cranial PSIs, mean, median, standard deviation, and interquartile range were calculated for each group. To visually inspect the accuracy of FFF fabricated PEEK PSIs, histograms were used to compare the mean differences (SD) and median differences (IQR) to the planned implant. This section of statistical analysis was performed in R statistical software (R Core Team 3.4.1, The R Foundation for Statistical Computing, Vienna, Austria).

## 3. Results

### 3.1. Optimized Nonthermal Printing Process Parameters

The conversion of percentage change in dimensions to the S/N ratio was carried out using the smaller-the-better quality characteristic. ANOVA and S/N ratio analysis were conducted to identify the optimum combination of process parameters. Table 4 displays the response values for the S/N ratio. The result concludes that the change in dimension was affected mostly by the infill pattern followed by the layer thickness, number of shells, and lastly, by the infill rate.

**Figure 5:**
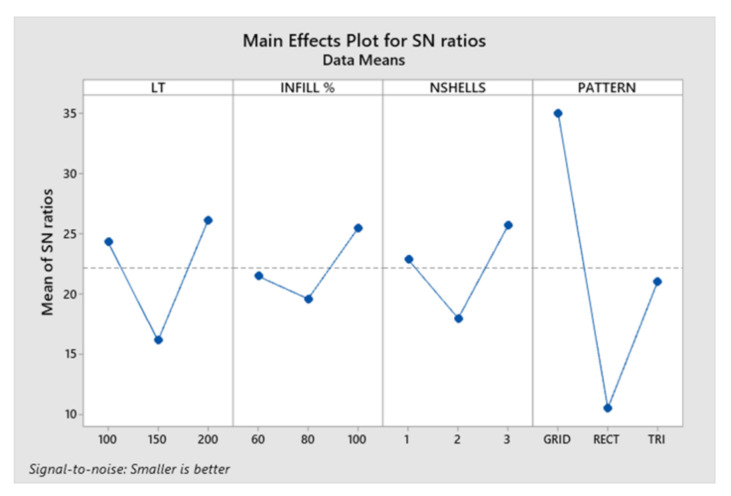
Main effects plot for signal-to-noise (S/N) ratio. LT, layer thickness; INFILL%, infill rate; NSHELLS, number of shells; PATTERN, infill pattern.

The main effects plot for S/N ratio are shown in Figure 5. The combined analysis of Table 3 and Figure 5 show that the combination of A_2_B_2_C_2_D_2_, i.e., layer thickness (A) of 150 μm, infill rate (B) of 80%, number of shells (C) as 2, and infill pattern (D) of rectilinear, were observed as the optimum parameters for minimum change in dimensions.

The results from ANOVA revealed that the number of shells had a significant influence on the change in dimensions. Additionally, ANOVA for transformed response displayed statistically significant interactions between layer thickness, infill rate, and infill pattern (*p* < 0.05). The contribution of the infill pattern was significantly higher (77.31%), followed by layer thickness (13.68%) and infill rate (8.70%).

### 3.2. Deviation Analysis and Dimensional Accuracy Assessment of FFF 3D Printed PEEK Cranial PSIs

**Figure 6:**
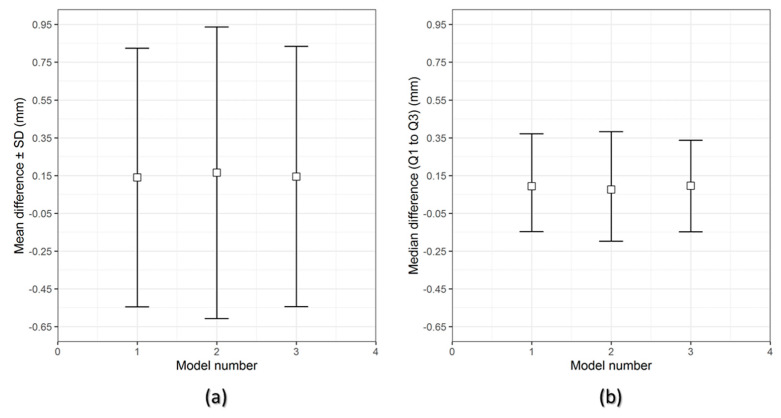
Descriptive data distribution for Group 1 PSIs, illustrating the difference between planned and FFF 3D printed PEEK PSIs (PSI model 1–3). (**a**) mean difference ± SD (mm) and (**b**) median difference (Q1 to Q3) (mm).

**Figure 7:**
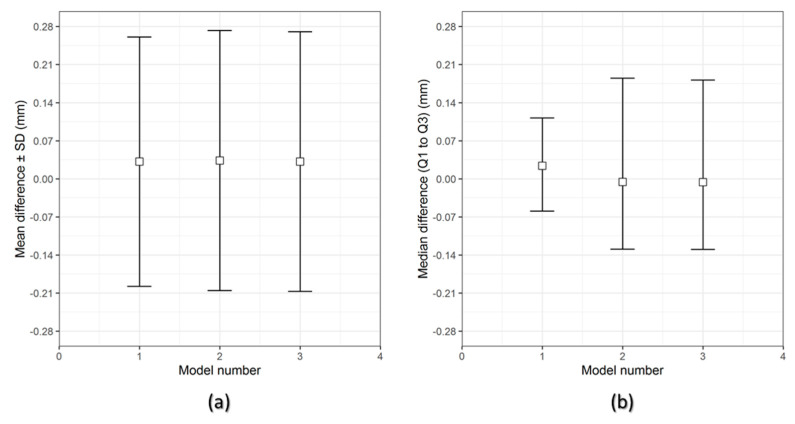
Descriptive data distribution for Group 2 PSIs, illustrating the difference between planned and FFF 3D printed PEEK PSIs (PSI model 1–3). (**a**) mean difference ± SD (mm) and (**b**) median difference (Q1 to Q3) (mm).

Figure 6 and Figure 7 show the overall descriptive data distribution for the difference between the planned (reference) and FFF 3D printed PEEK PSIs in Group 1 and Group 2, respectively.

In Group 1 PEEK PSIs, the comparison analysis revealed a mean difference ± SD of 0.731 ± 0.051 mm and a median difference (Q1 to Q3) of 0.704 (0.699–0.790) mm. On the other hand, the comparative analysis in Group 2 PSIs revealed a mean difference ± SD of 0.238 ± 0.006 mm and a median difference (Q1 to Q3) of 0.241 (0.232–0.242) mm. 

For the assessment of dimensional accuracy of FFF 3D printed PEEK cranial PSIs, the overall 3D deviations or RMS values were analyzed. Table 5 outlines the results of a quantitative dimensional accuracy assessment (RMS values). Higher the RMS value, more significant is the deviation error between two datasets and lower is the dimensional accuracy of the PSI. Higher deviations were observed in Group 1 as compared to Group 2 cranial PSIs. The highest RMS value in Group 1 PSIs was 0.790 mm, whereas in Group 2, the highest RMS value was 0.241 mm.

**Figure 8:**
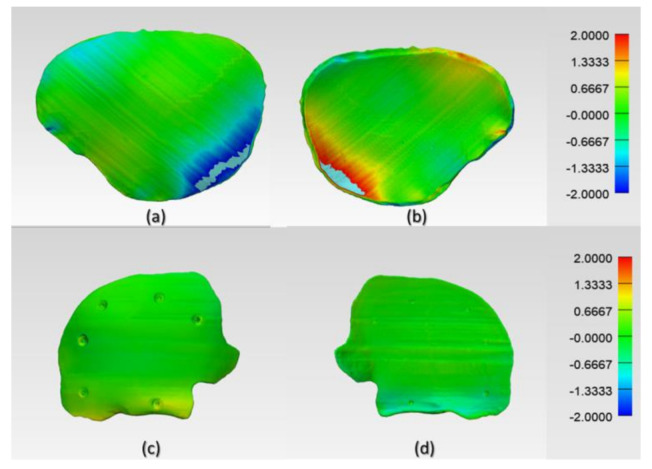
Color-coded deviation maps illustrating the areas of congruence or incongruence between planned and FFF 3D printed PEEK PSIs. Group 1 PSI: (**a**) squamous (outer) surface and (**b**) cerebral (inner) surface and Group 2 PSI: (**c**) squamous (outer) surface and (**d**) fronto-orbital (inner) surface.

The digitized surface meshes of FFF 3D printed PEEK PSIs were superimposed on the reference surface meshes of planned PSIs to generate a visual display of the location and magnitude of congruence or incongruence. Figure 8 shows the color deviation maps (3-matic medical 13.0, Materialise, Leuven, Belgium) generated for Group 1 and Group 2 PEEK PSIs. The blue areas of the color deviation map show a negative deviation, whereas the red-colored regions show a positive deviation.

**Figure 9:**
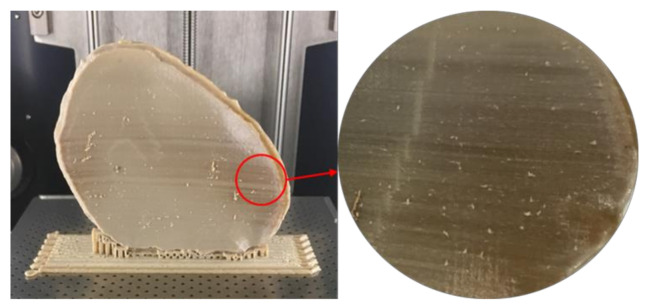
Signs of slight discoloration (dark-brownish areas) in Group 1 PSIs.

In Group 1, a slight negative deviation was observed on the squamous (outer) surface at the frontotemporal region of the PSI, whereas positive deviations were found on the cerebral (inner) surface at the anterolateral part of the PSI. In Group 2, a slight positive deviation was observed on the squamous (outer) surface at the supraorbital region, and negative deviation was seen on the orbital (inner) surface at the fronto-orbital margin of the PSI. On subjective evaluation, the deviations in Group 2 corresponded with the areas where the support structures were attached to the PSIs (Figure 3), whereas in Group 1 PSIs, these deviations corresponded with the areas which had signs of slight discoloration during the printing process (Figure 9). Moreover, horizontal lines, or the characteristic FFF stair-stepping effect, were more perceptible across the surface of Group 1 PSIs. The PSIs marginal fit was assessed subjectively on 3D printed cranial defect anatomical models by a consultant surgeon and was considered as acceptable.

## 4. Discussion

Personalized medicine has revolutionized the practice of modern medicine. With advancements in CAD and AM technologies, the use of customized implants with excellent cosmetics and functional results has now become widespread [22]. These technological improvements have led to a tremendous increase in the use of patient-specific alloplastic implants for cranioplasty applications. PEEK has been used in cranioplasty as a reliable alternative to other alloplastic materials [23,24]. Previous studies have shown the possibility of printing PEEK by FFF [17,25,26]; however, studies on FFF 3D printed PEEK cranial implants are limited. Therefore, to investigate the outcome of quality and clinical relevance of FFF 3D printing technology at POC manufacturing, the present study was conducted. We reported quantitative assessments of dimensional accuracy characteristics of FFF 3D printed PEEK PSIs using an in-house PEEK 3D printer.

The results of the dimensional accuracy in the present study revealed that Group 2 PSIs had comparatively lower deviations than Group 1 PSIs. In the illustrated Group 1 case, the highest RMS value was 0.790 mm, while in Group 2 case, the highest RMS value was 0.241 mm. The level of accuracy required in a PSI depends on the clinical application. According to our results, PSIs in Group 1 and Group 2 were within the acceptable accuracy range required in cranioplasty procedures, with overall 3D deviations under 2 mm [27]. Further analysis of the spatial distribution of variations revealed that the deviation pattern depended on the size and shape of the cranial defect, which was reflected in the PSIs fabricated (Figure 8). The more considerable deviations in Group 1 PSIs can be explained due to the more significant anatomical cranial defect with greater span of the curvature.

The results suggest that the dimensional characteristics of FFF 3D printed PEEK PSIs are a comprehensive consequence of a multitude of factors, including the thermal and nonthermal printing parameters of the 3D printer, the crystallinity of the parts, and the bonding interface between the printed layers. Group 1 PSIs displayed different color zones (Figure 9) compared to Group 2 PSIs. These color changes can be explained due to varying levels of crystallinity, i.e., dark-brownish areas have a more amorphous PEEK structure, whereas the lighter areas have a higher degree of crystallinity. Studies have shown that PEEK mechanical properties are influenced by the level of crystallinity of the material. Increasing the crystallinity can improve the elastic modulus and yield strength of the fabricated PEEK part [28]. Vaezi and Yang [29] found that heat management during the FFF 3D printing process and optimum heat distribution around the part are essential parameters to affect the level of crystallinity in the 3D printed PEEK object. Jin et al. [30] also showed that crystallinity in PEEK parts is influenced by the thermal processing conditions, such as the material cooling rate or thermal gradient. The amorphous regions in the printed parts can be optimized in the FFF printing process if the deposited materials are cooled down slowly or printed at a higher temperature to allow generation of crystalline PEEK structure [31].

**Figure 10:**
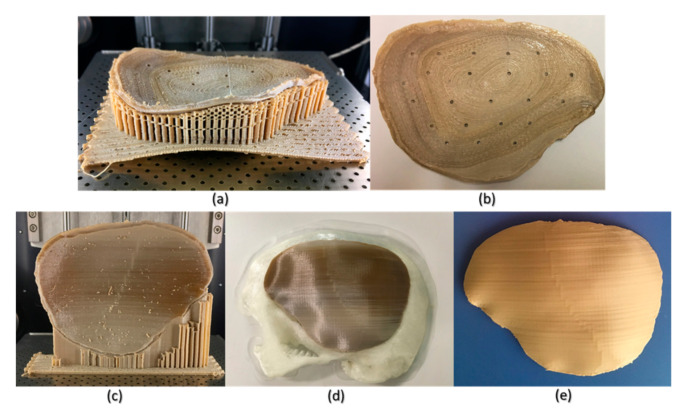
Illustrations of the FFF PEEK 3D printing issues in the cranial implants regarding different orientations. (**a**) horizontally printed cranial implant showing raft detachment/warping effect (in situ); (**b**) horizontally printed cranial implant displaying rough internal surface; (**c**) vertical printed cranial implant exhibiting different levels of crystallinity (in situ); (**d**) 3D printed skull biomodel with the vertically printed implant after support structure removal; and (**e**) annealed vertically printed cranial implant displaying no discolorations.

Although FFF seems like a simple process, the achievement of high efficiency and high-quality manufacturing results in PEEK printing presents significant challenges. The temperature of the illustrated FFF PEEK 3D printer used in our study was not user controlled; therefore, nonthermal printing parameters were tested to understand the dimensional characteristics. To further understand the issues with improper crystallinity, two additional cranial plates were printed; one in a horizontal build orientation and the other in a vertical build orientation positioned slightly away from the center of the build platform. We found that the test plate printed in a horizontal orientation had a very rough surface finish (Figure 10b) but with fewer regions of amorphous PEEK. The uneven fabrication lines were caused because of the combination of the building orientation and complex geometry of the cranial implant. Moreover, by the end of the printing process, warping or detachment of the implant from the printer’s build platform was noticed (Figure 10a). This effect can be explained because of the residual stress buildup that occurs during the printing process and the inability of the printer to maintain the required high processing temperatures consistently. In contrast, while the implant printed in a vertical orientation had a smoother surface finish, it also depicted different zones of crystallization (Figure 10b,c). From the support structure removal perspective, the parts printed in a vertical orientation provided a much faster support removal than horizontally oriented printed counterparts. Therefore, it can be inferred that part orientation and usage of support structures affects the surface finish and dimensional accuracy in complex anatomically shaped PSIs.

Several studies have reported that further postprocessing procedures such as annealing [28,32] could eliminate the dark amorphous regions caused due to irregular crystallization in PEEK parts. Figure 10d,e illustrates a PSI, before and after annealing postprocessing procedure. We noticed that the annealed cranial implant had an incomprehensible shrinkage and was, therefore, considered unfit for its clinical application. We, therefore, believe that although annealing helps to eliminate the amorphous regions and increases the mechanical strength of a part [33], it also results in marked dimensional deviations, especially in complex anatomically shaped cranial implants. However, to further comprehend whether the variations with annealing postprocessing procedure are influenced by the dimensions and contours of a cranial prosthesis, further studies are required. Nonetheless, the issues with recrystallization of amorphous regions and an additional requirement of high-temperature postprocessing procedures can limit the use of FFF 3D printed PEEK cranial implants for in-hospital manufacturing.

**Figure 11:**
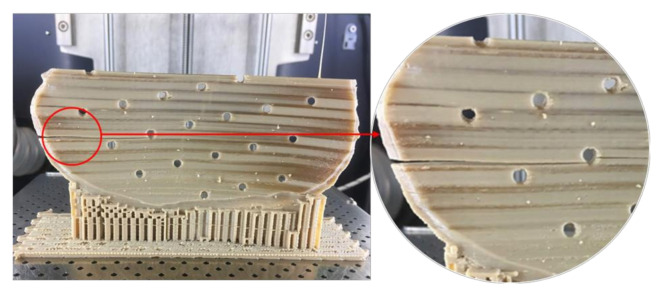
Structural failure in the PEEK cranial implant during the FFF 3D printing process.

Unlike Group 2, we printed the PSIs in Group 1 with no drainage holes. We resorted to this fabrication method, seeing the best-fit option for the illustrated PSI in our study. We learned that the orientation of the cranial implant profoundly influenced the fabrication feasibility of cranial implants with holes. As Group 1 PSIs were more extensive in dimensions than Group 2 PSIs, the fabrication of implant with drainage holes was possible only in a horizontal orientation, which resulted in a very rough surface finish. Moreover, printing the implant with holes in a vertical orientation resulted in plate breakage during the fabrication process (Figure 11). In FFF PEEK printing, the printed object attempts to accommodate these structural design effects, consequently leading to the initiation of internal stresses within the object. This results in a buildup of residual stresses, leading to crack propagation in the printed part. Due to the layer-by-layer fabrication method, each new layer is overlaid on top of the previous layer before material solidification from the melt occurs, resulting in volume shrinkage in the previous layer. The volume shrinkage contributes to weak interlayer bonding, and therefore, the structural failures are often confined to the interface between the layers [34]. All these effects invariably contribute towards a requirement of optimum thermal management during the PEEK printing process, along with a consideration of the principles of design for additive manufacturing.

Until now, PEEK PSIs have been manufactured by external MedTech companies. This production method sometimes takes several weeks and requires numerous meetings between clinicians and biomedical engineers. Furthermore, the expenses related to the manufacturing of customized PEEK cranial implants are high and depend on the defect size and shape [35]. For example, the average cost for the Group 1 and Group 2 PEEK PSIs procured from external companies is around 7000–10,000 € and 3000–4000 €, respectively. Nonetheless, the use of 3D printing technology in hospitals would be very advantageous. This could significantly reduce the production lead times and treatment times, thereby increasing patients’ satisfaction and surgical outcomes.

As the AM of PSIs is gradually moving towards in-house or POC manufacturing, clinicians need to understand the various factors that can affect the quality of the fabricated implant. As per the guidelines published in “Additively Manufactured Medical Products—The FDA Perspective,” like any external service provider, hospital-based 3D printing set-ups should provide the same efficacy and manufacturing quality for medical devices [36]. Besides, organizations such as the American Society for Testing and Materials (ASTM) International, the International Organization for Standardization (ISO), and the Association for the Advancement of Medical Instrumentation (AAMI) have proposed standard technical consensus for PEEK medical devices [37]. Furthermore, standardized operational measures such as quality management protocols should be implemented in the hospital environment to assess whether the intended 3D printed part conforms to its clinical application [38]. One aspect of these protocols is part verification, which was analyzed in this study. Although the dimensional accuracy of PSIs fabricated in both the groups were within the clinically acceptable range; however, attention must be paid to the temperature control during the printing process to ensure that it is well regulated to fabricate implants of consistent crystallinity.

AM processing of PEEK thermoplastic polymer for the fabrication of large-sized, complex cranial implants presents significant challenges due to the limitations associated with large thermal gradients, residual stress buildup, and the inability of the 3D printer to provide the required ambient temperatures consistently. A beta version of an updated software from the FFF PEEK 3D printer’s manufacturer, which promises a layer-by-layer incremental increase in the airflow temperature, is in the developmental stage. Therefore, these results will need to be revisited to access the improved performance of the FFF PEEK printing process for medical implants at the POC manufacturing. Another aspect that needs attention is the anisotropic behavior of the FFF 3D printed PEEK cranial implants. Due to the layer-by-layer fabrication method, the specific anisotropic performance needs to be tested in future experiments addressing the biomechanical properties of the FFF 3D printed PEEK cranial implants.

## 5. Conclusions

With 3D printing laboratories in hospitals worldwide, the production of anatomical biomodels has become more tangible. However, 3D printing of PSIs at the POC is still rare. It can be inferred from this study that although POC manufacturing has a vast potential for PEEK PSIs in craniofacial reconstructions, however, technological aspects are still in the nascent stage. Further advancement in this technology will open up enormous scope for innovation and future development in various surgical applications.

## Figures and Tables

**Table 1:** Technical specifications of the fused filament fabrication (FFF) polyetheretherketone (PEEK) three-dimensional (3D) printer.

Parameter	Specifications
Number of extruders	1
Nozzle diameter	0.4 mm
Build platform	130 mm × 130 mm × 120 mm
Print head temperature	Up to 540 °C
Controlled airflow temperature	Up to 200 °C
Print bed material	316L stainless steel
Machine operation software	Apium control software

**Table 2:** FFF PEEK 3D printing process parameters and levels selected for the experiment.

Process Parameter	Symbol	Unit	Level 1	Level 2	Level 3
Layer thickness	A	μm	100	150	200
Infill rate	B	%	60	80	100
Number of shells	C	–	1	2	3
Infill pattern	D	–	Grid	Rectilinear	Triangular

**Table 3:** Taguchi L9 (3^4^) orthogonal array generated for the experiment.

Experiment Number		Control Factors		
	Layer Thickness	Infill Rate	Number of Shells	Infill Pattern
1	1	1	1	1
2	1	2	2	2
3	1	3	3	3
4	2	1	2	3
5	2	2	3	1
6	2	3	1	2
7	3	1	3	2
8	3	2	1	3
9	3	3	2	1

**Table 4:** Response values for signal-to-noise (S/N) ratio.

Parameters	Symbol	Level 1	Level 2	Level 3	Rank
Layer thickness	A	24.29	16.10	26.10	2
Infill rate	B	21.46	19.57	25.46	4
Number of shells	C	22.84	17.95	25.69	3
Infill pattern	D	35.04	10.48	20.97	1

**Table 5:** Quantitative assessment of dimensional accuracy of FFF 3D printed PEEK patient-specific implants (PSIs) regarding root mean square (RMS) values (mm).

Group 1	RMS (mm)	Group 2	RMS (mm)
PSI 01	0.699	PSI 01	0.232
PSI 02	0.790	PSI 02	0.241
PSI 03	0.704	PSI 03	0.241

## Abbreviations

3D	Three-dimensional
AAMI	Association for the Advancement of Medical Instrumentation
AM	Additive manufacturing
ANOVA	Analysis of variance
ASTM	American Society for Testing and Materials
CAD	Computer-aided design
CAM	Computer-aided manufacturing
CD	Change in the dimensions
CT	Computed Tomography
DICOM	Digital Imaging and Communications in Medicine
DOE	Design of experiments
EOS	Electro-Optical Systems
FFF	Fused filament fabrication
ICP	Iterative closest point
INFILL %	Infill rate
ISO	International Organization for Standardization
IQR	Interquartile range
LT	Layer thickness
MIMICS	Materialise Interactive Medical Image Control System
MSD	Mean square deviation
NSHELLS	Number of shells
PATTERN	Infill pattern
PEEK	Polyetheretherketone
POC	Point-of-care
PSIs	Patient-specific implants
RMS	Root mean square
SD	Standard deviation
SLS	Selective laser sintering
S/N	Signal-to-noise
STL	Standard Tessellation Language
